# Diabetes mellitus as a risk factor for chemotherapy-induced peripheral neuropathy: a meta-analysis

**DOI:** 10.1007/s00520-021-06321-7

**Published:** 2021-06-03

**Authors:** Jialin Gu, Hong Lu, Chen Chen, Zhancheng Gu, Miao Hu, Ling Liu, Jialin Yu, Guoli Wei, Jiege Huo

**Affiliations:** 1 Department of Oncology, Affiliated Hospital of Integrated Traditional Chinese and Western Medicine, Nanjing University of Chinese Medicine, Nanjing, 210028 Jiangsu China; 2 Graduate School, Nanjing University of Chinese Medicine, Nanjing, 210046 Jiangsu China; 3 Department of Oncology, Changshu Hospital Affiliated To Soochow University, First People’s Hospital of Changshu City, Suzhou, 215500 Jiangsu China; 4 Department of Oncology, Yancheng Hospital Affiliated to Nanjing University of Chinese Medicine, Yancheng, 224001 Jiangsu China; 5 Department of Oncology, Jiangsu Province Academy of Traditional Chinese Medicine, Nanjing, 210028 Jiangsu China

**Keywords:** Chemotherapy-induced peripheral neuropathy (CIPN), Diabetes mellitus (DM), Risk, Meta-analysis

## Abstract

**Background:**

To identify the association between diabetes mellitus (DM) and the risk of chemotherapy-induced peripheral neuropathy (CIPN) through a systematic review and meta-analysis.

**Methods:**

An electronic literature search was conducted in PubMed, Embase, Web of Science, the Wanfang database, the VIP Journals database (CQVIP), the China National Knowledge Infrastructure (CNKI) database, and the China Biology Medicine database (Sinomed) between January 2010 and January 2021. Articles were included if they investigated CIPN and DM. Stata 15.1 was used to analyze the data.

**Results:**

We examined 8923 cancer patients from 25 studies comprising 9 cohort studies and 16 case–control studies. Meta-analysis showed that there was a statistically significant positive correlation between DM and CIPN (odds ratio [OR] = 1.60, 95% confidence interval [CI] = 1.38–1.85, *P* < 0.001). Egger’s test (*P* = 0.824) showed no evidence of publication bias. The positive associations did not significant differ by study type, study quality, evaluation instrument, and type of antineoplastic drug. Omission of any single study had little effect on the combined risk estimate. Little evidence of heterogeneity was observed.

**Conclusion:**

This meta-analysis provides evidence of a significant positive association between DM and risk of CIPN. Furthermore, a more detailed evaluation is warranted for cancer patients with diabetes when they are treated with antineoplastic drugs that have the potential to cause peripheral neuropathy.

**Supplementary Information:**

The online version contains supplementary material available at 10.1007/s00520-021-06321-7.

## Introduction

With the gradual prolongation of the survival time of cancer patients, attention should increasingly focus on the long-term toxicity associated with cancer treatment because of its potential to affect the quality of life of cancer patients [1]. Chemotherapy-induced peripheral neuropathy (CIPN), which can lead to permanent symptoms and disability in cancer survivors, is a prominent complication associated with this long-term toxicity [2]. CIPN is a frequent side effect of several commonly used antineoplastic agents, including platinum-based drugs (cisplatin, carboplatin, and oxaliplatin), taxanes (paclitaxel and docetaxel), vincristine, and eribulin, all of which are widely used as therapies for a variety of cancers [3]. Studies have shown that the incidence of CIPN ranges from 19 to more than 85% [4, 5]. Long-term follow-up results suggest that CIPN symptoms may persist for several years or even a lifetime after the cessation of chemotherapy, seriously affecting the quality of life of patients [6].

The most common symptoms of CIPN are sensory symptoms, such as pain, numbness, and tingling. However, some patients may have difficulties in fine motor coordination, sensory ataxia, and autonomic dysfunction [7]. Although numerous clinical studies have been conducted on the prevention and treatment of CIPN, none has provided conclusive evidence for a clinically beneficial agent in the treatment of CIPN, except for duloxetine, which is currently recommended for the treatment of painful neuropathy [8]. Therefore, understanding the risk factors for this side effect of chemotherapy is critical for preventing severe CIPN and may help guide further research and treatment. Recent studies have shown that drug cumulative dose is the most important influencing factor and accurate predictor of all CIPN [7, 9]. Other possible factors include the duration of drug infusion, baseline neuropathy, age, sex, smoking history, renal dysfunction (low creatinine clearance rate), metabolic-lifestyle factors, and genetic predisposition [7, 9–13].

Diabetes mellitus (DM) is one of the most important, chronic, noncommunicable diseases worldwide. Many cancer patients have a history of DM. Diabetic peripheral neuropathy (DPN) is a common chronic complication in diabetic patients and its clinical manifestations are similar to those of CIPN. The relationship between DM and CIPN is controversial. Although preexisting DPN is considered to be a risk factor for CIPN, it is unclear whether the incidence and severity of the latter are greater in patients with diabetes who do not have peripheral neuropathy symptoms at baseline when they receive chemotherapy [14]. Therefore, we conducted a meta-analysis to quantitatively evaluate the association between CIPN and DM among cancer patients.

## Methods

### Search strategy

We conducted a comprehensive search in PubMed, Embase, and Web of Science (WOS). Chinese databases, including the Wanfang database, VIP Journals database (CQVIP), and China National Knowledge Infrastructure (CNKI) database, were also searched in order to expand the scope of retrieval. We obtained all studies published between January 2010 and January 2021 that reported on DM and CIPN using the Medical Subject Headings (MeSH) terms “chemotherapy” or the text word terms “antineoplastic agents,” “oxaliplatin,” “paclitaxel,” “docetaxel,” “vincristine,” “bortezomib,” “thalidomide,” or “platinum”; the MeSH term “diabetes mellitus” or the text word terms “diabetes complications,” “IDDM,” “NIDDM,” “MODY,” “T1DM,” or “T2DM”; and the MeSH term “neurotoxicity” or the text word terms “neuropathy,” “neuropathic,” or “nerve.” Multiple combinations of the above search terms were used. There was no limit to the use of the word term “peripheral” to avoid omissions as much as possible. Only studies that were published in the English or Chinese language were considered.

This study conformed to the PRISMA guidelines (the Preferred Reporting Items for System Reviews and Meta-Analysis) statement.

### Study selection

Our primary research question concerned the role of DM in the development of CIPN. Therefore, we looked for longitudinal studies that contained both exposure factors (DM) and clinical outcomes (incidence of CIPN) and comprised both diabetic and nondiabetic patients with or without CIPN. Case–control studies and cohort studies were included. The titles, abstracts, and subsequent full text of the retrieved publications were screened by two independent reviewers. Any disagreement between the reviewers was resolved by a third independent reviewer. Inclusion criteria were original case–control or cohort studies with outcome indicators that included the incidence of CIPN in diabetic or nondiabetic patients. Reviews, conference abstracts, case reports, editorials, letters to editors, repetitive publications, and studies for which data were unavailable were excluded. Endnote (V9.3.3, Clarivate Analytics) was used for literature screening and management. References in the literature that met the inclusion criteria were manually screened to prevent omissions.

### Data extraction

Two independent reviewers extracted the following data from studies that met the inclusion criteria after duplicate checking: first author, time of publication, type of research, study population, grade of CIPN, antineoplastic drugs studied, outcome measure, and type of cancer.

### Quality assessment

The Newcastle–Ottawa scale (NOS) was used to evaluate the quality of the included literature. Studies were judged on three aspects: selection, comparability, and exposure; under each aspect, there were several items for researchers to score, with a total maximum score of nine. Except for comparability (two points), the highest score for each of the other items was one point. Scores ranging from zero to three, four to six, and seven to nine represented low-, medium-, and high-quality studies, respectively. This scale is widely used in the quality assessment of nonrandomized studies and is suitable for use in case–control and cohort studies. The quality of the included studies was evaluated by two independent reviewers. A second review of the studies for which there was disagreement was conducted by the third reviewer. The quality of the studies was assessed using an adjusted NOS scale. Studies with scores greater than five were included in the subsequent meta-analyses. The details are described in Tables S1 and S2.

### Statistical analysis

A meta-analysis of the data extracted from the studies that met the quality assessment was performed using Stata V15.1. Because the outcome indicator (incidence of CIPN) for this study was a bicategorical variable, odds ratios (ORs) and 95% confidence intervals (CIs) were used as the combined effect measures. Heterogeneity among studies was assessed using the chi-squared and I-squared (*I*^2^) tests. Random-effects models were used for pooling the results of different studies when *P* < 0.1 or *I*^2^ > 50%; otherwise, fixed-effects models were used for combined data. The risk of publication bias was calculated using Egger’s test and significant publication bias was determined at *P* < 0.05. Subgroup analysis was also conducted for important variables. The following potential influential stratified factors were considered: study type, study quality, evaluation instrument, and type of antineoplastic drug (mainly oxaliplatin and taxane). Interaction and heterogeneity tests were performed to detect the influence of each stratified factor on the relationship between DM and CIPN. In addition, a sensitivity analysis was constructed in which one study at a time was excluded and others were analyzed to estimate whether the results would be significantly affected by certain studies.

## Results

### Literature selection

**Fig. 1:**
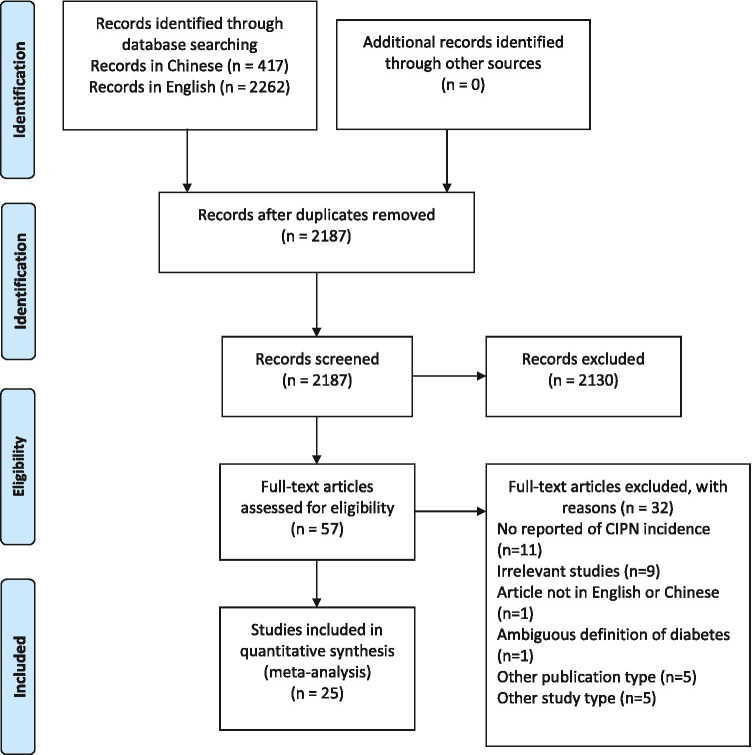
PRISMA flow chart of study identification and selection

The search identified 417 publications derived from Chinese databases (Wanfang, CQVIP, and CNKI), and 2262 derived from PubMed, Embase, and WOS. Of these, 2187 were unique. Based on the titles and abstracts of all the articles screened by the reviewers, 2131 articles were excluded because they did not meet the inclusion criteria. After screening the full text of the 57 remaining studies that met the inclusion criteria, 25 studies were finally included in the meta-analysis as they clearly reported the incidence or severity of CIPN. Figure 1 shows the PRISMA flow chart of study identification and selection.

### Quality assessment

The quality of the articles was independently assessed by at least two reviewers. All the 25 articles scored 5 points or more on the NOS scale, indicating a moderate to good overall study quality; 22 of the 25 studies were of high quality (scored 7 to 9 points). One study [15] reported the incidence of CIPN under two treatment regimens. Therefore, a total of 26 studies were included in the meta-analysis, including 9 cohort studies and 17 case–control studies. Nine of the 22 studies [15–23] reported a positive association between diabetes and CIPN, whereas the others reported no association. Table 1 shows the characteristics of the included studies. The quality assessment results of all the studies are shown in Tables S3 and S4.

**Table 1:** Study characteristics

First author	Year	Type of study	*N*	Grade of CIPN	Main types of antineoplastic drugs	Outcome measure	Type of cancer
Molassiotis A	2019 [24]	Cohort	255	≥ 1	Platinum, taxane	NCI-CTCAE	Breast, lung, ovarian, gastrointestinal, head & neck, and urinary tract cancers
Chen C	2019 [16]	Cohort	60	≥ 1	Oxaliplatin	WHO standard	Colorectal, gastric, and esophageal cancers
Hertz DL	2018 [25]	Cohort	60	Self-report	Cisplatin	EORTC QLQ-CIPN20	Breast cancer
Gaballah A	2018 [17]	Case–control	250	≥ 1	Platinum, taxane	NCI-CTCAE	NA
Yamaguchi K	2018 [26]	Cohort	60	≥ 2	Oxaliplatin	NCI-CTCAE	Gastric cancer
Dolan ME	2017 [27]	Cohort	680	Self-report	Cisplatin	EORTC QLQ-CIPN20	germ cell cancer
Song SJ	2017 [28]	Case–control	1516	≥ 2	Taxane	Cancer pain management guideline, 6th edition	Breast cancer
Hershman DL	2016 [23]	Case–control	1401	≥ 2	Multiple chemotherapy regimens	NCI-CTCAE	lung, prostate, breast, head and neck, bladder, and ovarian cancers
Bao T	2016 [29]	Case–control	296	≥ 1	Taxane	Cancer-related symptom rating scales	Breast cancer
Tanishima H	2016 [30]	Case–control	47	≥ 1	Oxaliplatin	NCI-CTCAE	Colorectal cancer
Pereira S	2016 [31]	Cohort	296	≥ 1	Taxane	NCI-CTCAE	Breast cancer
Wang YQ	2016 [18]	Case–control	225	≥ 2	Oxaliplatin, paclitaxel, vincristine	NCI-CTCAE	Colorectal, gastric, esophageal, lung, and ovarian cancers; lymphoma
Shahriari-Ahmadi A	2015 [32]	Case–control	130	≥ 1	Oxaliplatin	NCI-CTCAE	Colorectal cancer
Kus T^a^	2015 [15]	Case–control	270	≥ 1	Taxane	NCI-CTCAE	NA
Kus T^a^	2015 [15]	Case–control	104	≥ 1	Taxane, platinum	NCI-CTCAE	NA
de la Morena Barrio P	2015 [19]	Case–control	129	≥ 1	Paclitaxel	NCI-CTCAE	Breast cancer
Ding XF	2015 [20]	Cohort	94	≥ 1	Paclitaxel	Self-definition	Prostate, breast, ovarian, bladder, lung, and cervical cancers
Johnson C^b^	2015 [21]	Case–control	735	≥ 1	Platinum, taxane	NCI-CTCAE	Lung cancer
Eckhoff L	2014 [33]	Cohort	150	≥ 2	Docetaxel	NCI-CTCAE	Breast cancer
Xue YJ	2013 [22]	Cohort	80	≥ 1	Paclitaxel	Levi	Breast, lung, esophageal, gastric, ovarian, and uterine cancers
Wang XY	2013 [34]	Case–control	171	≥ 2	Oxaliplatin	Levi	Colorectal cancer
Hashimoto N	2012 [35]	Case–control	48	≥ 2	Bortezomib	NCI-CTCAE	Multiple myeloma
Kawakami K	2012 [36]	Case–control	50	≥ 3	Paclitaxel, carboplatin	NCI-CTCAE	Non-small cell lung cancer
Vincenzi B	2012 [37]	Case–control	169	≥ 2	Oxaliplatin	NCI-CTCAE	Colorectal cancer
Uwah AN	2012 [38]	Case–control	62	≥ 2	Oxaliplatin	NCI-CTCAE	Colorectal cancer
Ramanathan RK	2010 [39]	Case–control	1585	≥ 1	Oxaliplatin	NCI-CTCAE, oxaliplatin-specific neurotoxicity scale	Colorectal cancer

*NCI-CTCAE* National Cancer Institute-Common Toxicity Criteria for Adverse Events; *EORTC QLQ-CIPN20* European Organisation for Research and Treatment of Cancer Quality of Life Questionnaire Chemotherapy-Induced Peripheral Neuropathy; *CIPN* chemotherapy-induced peripheral neuropathy

^a^The incidence of CIPN was clearly distinguished between the two treatment schemes

^b^Patients with unknown CIPN status were excluded from statistical analysis

### Correlation between DM and CIPN

**Fig. 2:**
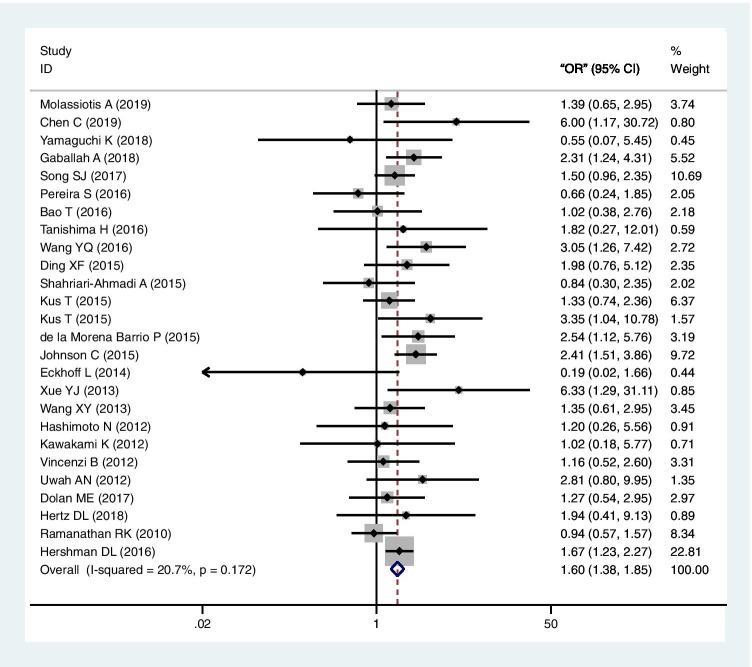
Forest plot of the meta-analysis of the association between diabetes mellitus and chemotherapy-induced peripheral neuropathy

All 26 studies explored the association between CIPN and DM. Figure 2 shows the forest plot of these studies in the meta-analysis (a total of 8923 patients). Because the heterogeneity test showed that there was no statistical heterogeneity among the studies (*I*^2^ = 20.8%, *P* = 0.172), a fixed-effects model was used to analyze the outcome indicators. The results showed that there was a statistically significant positive correlation between DM and CIPN (OR = 1.60, 95% CI = 1.38–1.85, *P* < 0.01). Egger’s test (*P* = 0.824) showed no evidence of publication bias.

### Subgroup and sensitivity analysis

Table 2 shows the subgroup analyses for study type, study quality, evaluation instrument, and type of antineoplastic drug. A positive association between DM and CIPN was observed in all subgroup analyses. Low or moderate of heterogeneity was identified within any subgroup. Notably, in the antineoplastic drug type subgroup analysis, eight studies reported oxaliplatin-induced neuropathy, while eight reported taxane-induced neuropathy. The results showed that the risk of CIPN in diabetic cancer patients treated with taxane-based drugs (OR = 1.47, 95% CI = 1.11–1.93) was higher than that in nondiabetic cancer patients. The same result could also be observed in the oxaliplatin subgroup (OR = 1.19, 95% CI = 0.86–1.65). The association between DM and CIPN risk was not significantly modified by antineoplastic drugs (*P* for interaction = 0.81). The combined ORs of overall risk estimates were consistent and without apparent fluctuation based on the sensitivity analyses, with a range from 1.53 (95% CI = 1.31–1.78) to 1.68 (95% CI = 1.44–1.95).

**Table 2:** Subgroup analyses of the association between diabetes and CIPN for study type, study quality, evaluation instrument and type of antineoplastic drug

Subgroup	No. of studies	No. of patients		OR (95% CI)	*P* heterogeneity	*I*^2^ (%)	*P* interaction
		DM + , n	DM − , n				
Total	26	1605	7318	1.60 (1.38–1.85)	0.172	20.8	
Type of study							0.88
Cohort	9	364	1371	1.44 (0.98–2.11)	0.107	39.1	
Case–control	17	1241	5947	1.63 (1.39–1.91)	0.320	11.4	
Study quality							0.60
High	23	1239	6775	1.50 (1.28–1.75)	0.304	11.5	
Moderate	3	366	543	2.48 (1.65–3.72)	0.458	0	
Evaluation instrument							0.87
Others	10	408	3394	1.50 (1.23–1.83)	0.284	17.3	
NCI-CTCAE	16	1197	3924	1.72 (1.39–2.15)	0.179	24.3	
Type of antineoplastic drug							0.81
Oxaliplatin only^a^	8	284	1992	1.19 (0.86–1.65)	0.369	7.9	
Taxane only^b^	8	478	2353	1.47 (1.11–1.93)	0.114	39.8	

^a^Eight studies reported the association between DM and oxaliplatin-induced neuropathy

^b^Eight studies reported the association between DM and taxane-induced neuropathy

*DM* diabetes mellitus; *CIPN* chemotherapy-induced peripheral neuropathy; *OR* odds ratio; *CI* confidence interval; *NCI-CTCAE* National Cancer Institute-Common Terminology Criteria for Adverse Events

## Discussion

Chemotherapy is an important treatment method for malignant tumors. Some antineoplastic drugs are irreplaceable for patients receiving postoperative adjuvant therapy, such as oxaliplatin for colorectal cancer and taxanes for breast cancer. However, many patients will develop peripheral neuropathy when administered these kinds of drugs, which affects quality of life and treatment compliance [6, 40]. No effective agents are currently recommended for the prevention of CIPN, while duloxetine has shown limited efficacy in clinical studies [41]. Consequently, preventing and treating CIPN remains clinically challenging. The identification of risk factors for CIPN enables clinicians to assess patients more accurately and pay more attention to it, whether in applying antineoplastic drugs or combining other therapies.

Neuropathy is also a common complication of DM, with up to 50% of diabetic patients developing peripheral neuropathy with disease progression [39]. Many cancer patients have a history of DM, and determining whether DM is a risk factor for CIPN is of importance in clinical practice. Our meta-analysis suggested that there was a positive correlation between DM and CIPN and the association was neither significantly modified by study quality, evaluation instrument, or type of antineoplastic drug nor substantially affected by any single study based on the results from our subgroup and sensitivity analyses.

A significant, robust, and positive association was found between DM and CIPN in all subgroups, except in the oxaliplatin subgroup. Although the mechanisms that lead to CIPN may differ between taxane and platinum, the nonsignificant association in oxaliplatin subgroup was likely due to fewer relevant studies and smaller number of sample size (*n* = 284) and, hence, insufficient statistical power. It is still important to note that different chemotherapeutic drugs affect distinct components of the nervous system. Both oxaliplatin and paclitaxel, as well as other agents, can cause DRG, axonal, and axonal component damage; however, paclitaxel and vincristine can also affect distal nerve terminals [42, 43]. Specifically, oxaliplatin and paclitaxel can cause mitochondrial dysfunction and oxidative stress injury in peripheral nerves. Paclitaxel can block axonal transport by inhibiting tubulin hydrolysis and interfering with normal axonal microtubule dynamics, while oxaliplatin can cause the abnormal function of ion channels, such as voltage-gated sodium channels, voltage-gated potassium channels, voltage-gated calcium channels, and transient receptor potential channels [9, 44, 45]. In general, paclitaxel- and other drug-induced peripheral neuropathies are similar to DPN in terms of mechanism and symptoms. Furthermore, most of the studies did not assess whether patients had DPN, especially mild symptoms, and did not record the duration of the DM. The assessment of DPN is different from that of CIPN, and the occurrence of DPN is closely related to the duration of this disease [39]. Consequently, it is impossible to know how many diabetic patients in these studies developed DPN, symptomatic, or otherwise. Previous study has reported that there was an association only in patients with complications of DM (i.e., PN from DM) and not in patients without complications [23]. Our meta-analysis preferred to show a significant association between DM and CIPN, regardless of the presence of comorbidities. However, such a conclusion may need to be further supported by additional studies on multivariables of DPN.

Given that DM is a high-risk factor for CIPN, a more detailed evaluation should be undertaken for cancer patients with diabetes when they are treated with chemotherapeutic agents that may cause peripheral neuropathy. First, the presence of diabetes and the duration of diabetes, especially the presence of DPN, should be fully evaluated before chemotherapy. If DPN is present, more intense monitoring for CIPN should be performed when using potentially neurotoxic drugs, and these patients could be recommended for clinical trials of CIPN prevention therapies. Second, when patients use drugs that can affect blood glucose, such as dexamethasone and glucose, attempts should be made to maintain blood glucose levels within the normal range during chemotherapy to reduce the possibility of the occurrence of CIPN and DPN.

This review had some limitations. First, although the quality assessment showed that most of the studies were of high quality, some studies nevertheless had a mild sample size, leading to potential bias. Second, most of the eligible studies were retrospective, and confounding factors may have biased the results. Third, there was no standardized definition of the incidence and grade of CIPN as the primary study outcome.

## Conclusions

Our findings have important clinical implications; CIPN remains a common side effect of chemotherapy. Controversies continue regarding the effects of DM and CIPN risk. We conducted a meta-analysis of these controversial studies, enhancing the ability to detect associations and providing more reliable estimates. Taken together, this study provides evidence of a significant positive association between DM and risk of CIPN. Furthermore, a more detailed evaluation is warranted for cancer patients with diabetes when they are treated with antineoplastic drugs that have the potential to cause peripheral neuropathy.

## Supplementary Information

Supplementary 1 Table S1: Adjusted Newcastle–Ottawa scale (NOS) for the cohort studies (PDF 64 kb)

Supplementary 2 Table S2: Adjusted Newcastle–Ottawa scale (NOS) for the case-control studies (PDF 60 kb)

Supplementary 3 Table S3: Adjusted Newcastle–Ottawa scale (NOS) scores for the cohort studies (PDF 53 kb)

Supplementary 4 Table S4: Adjusted Newcastle–Ottawa scale (NOS) scores for the case-control studies (PDF 51 kb)

Supplementary 5 Table S5: PRISMA 2009 Checklist (PDF 95 kb)

## Data Availability

The data sets supporting the results of this article are included within the article and its additional files.
