# Supplementary material for: Diabetes mellitus as a risk factor for chemotherapy-induced peripheral neuropathy: a meta-analysis
Source: Support Care Cancer. 2021 Jun 3;29(12):7461–9. doi: 10.1007/s00520-021-06321-7 (PMC8550712; doi:10.1007/s00520-021-06321-7)
Supplement: Supplementary file 1 — Table S1: Adjusted Newcastle–Ottawa scale (NOS) for the cohort studies (PDF 64 kb) [file 520_2021_6321_MOESM1_ESM.pdf]

NEWCASTLE - OTTAWA QUALITY ASSESSMENT SCALE
COHORT STUDIES
Review: diabetes and chemotherapy-induced peripheral neuropathy (CIPN)

<u>Note</u>: A study can be awarded a maximum of one star for each numbered item within the Selection and Outcome categories. A maximum of two stars can be given for Comparability

Selection

1) <u>Representativeness of the exposed cohort</u>
   a) truly representative of the general population *
   b) somewhat representative of the general population *
   c) selected group of users eg nurses, volunteers
   d) no description of the derivation of the cohort

2) <u>Selection of the non exposed cohort</u>
   a) drawn from the same community as the exposed cohort *
   b) drawn from a different source
   c) no description of the derivation of the non exposed cohort

3) <u>Ascertainment of exposure</u>
   a) secure record (eg surgical records) *
   b) structured interview *
   c) written self report
   d) no description

4) <u>Demonstration that outcome of interest was not present at start of study (no CIPN at start of study)</u>
   a) yes *
   b) no

Comparability

5) <u>Comparability of cohorts on the basis of the design or analysis</u>
   a) study controls for cumulated dose of antineoplastic agents *
   b) study controls for age, sex, type of antineoplastic agents, duration of treatment, race, type of cancer, Eastern Cooperative Oncology Group (ECOG) performance status, etc. *

Outcome

6) <u>Assessment of outcome</u>
   a) independent blind assessment (eg. doctor's diagnosis, objective measurements) *
   b) record linkage *
   c) self report
   d) no description

7) <u>Was follow-up long enough for outcomes to occur</u>
   a) yes (majority of population at least four cycles of treatment) *
   b) no

8) <u>Adequacy of follow up of cohorts</u>
   a) complete follow up - all subjects accounted for *
   b) subjects lost to follow up unlikely to introduce bias - small number lost - > 80 %

follow up, or description provided of those lost, proving a non-selective loss to follow up *

c) follow up rate < 80% and no description of those lost, or a selective loss to follow up

d) no statement
