# Supplementary material for: Diabetes mellitus as a risk factor for chemotherapy-induced peripheral neuropathy: a meta-analysis
Source: Support Care Cancer. 2021 Jun 3;29(12):7461–9. doi: 10.1007/s00520-021-06321-7 (PMC8550712; doi:10.1007/s00520-021-06321-7)
Supplement: Supplementary file 2 — Table S2: Adjusted Newcastle–Ottawa scale (NOS) for the case-control studies (PDF 60 kb) [file 520_2021_6321_MOESM2_ESM.pdf]

NEWCASTLE - OTTAWA QUALITY ASSESSMENT SCALE
CASE CONTROL STUDIES
Review: diabetes and chemotherapy-induced peripheral neuropathy (CIPN)

Note: A study can be awarded a maximum of one star for each numbered item within the Selection and Exposure categories. A maximum of two stars can be given for Comparability.

Selection

1) Is the case definition adequate?
   a) yes, with independent validation (eg. doctor's diagnosis, reference tot primary record source) *
   b) yes, eg record linkage or based on self reports
   c) no description

2) Representativeness of the cases
   a) consecutive or obviously representative series of cases *
   b) potential for selection biases or not stated

3) Selection of Controls
   a) community controls (same community as cases) *
   b) hospital controls
   c) no description

4) Definition of Controls
   a) no history of disease (endpoint) *
   b) no description of source

Comparability

5) Comparability of cases and controls on the basis of the design or analysis
   a) study controls for cumulated dose of antineoplastic agents *
   b) study controls for age, sex, type of antineoplastic agents, duration of treatment, race, type of cancer, Eastern Cooperative Oncology Group (ECOG) performance status, etc. *

Exposure

6) Ascertainment of exposure
   a) secure record (eg. doctor's diagnosis, objective measurements) *
   b) structured interview where blind to case/control status *
   c) interview not blinded to case/control status
   d) written self report or medical record only
   e) no description

7) Same method of ascertainment for cases and controls

   a) yes *
   b) no

8) Non-Response rate
   a) same rate for both groups *
   b) non respondents described
   c) rate different and no designation
