# Supplementary material for: Diabetes mellitus as a risk factor for chemotherapy-induced peripheral neuropathy: a meta-analysis
Source: Support Care Cancer. 2021 Jun 3;29(12):7461–9. doi: 10.1007/s00520-021-06321-7 (PMC8550712; doi:10.1007/s00520-021-06321-7)
Supplement: Supplementary file 3 — Table S3: Adjusted Newcastle–Ottawa scale (NOS) scores for the cohort studies (PDF 53 kb) [file 520_2021_6321_MOESM3_ESM.pdf]

Table S3 Adjusted Newcastle–Ottawa scale (NOS) scores for the cohort studies

Name	Year	Selection				Comparability	Outcome		
		Question 1	Question 2	Question 3	Question 4	Question 5	Question 6	Question 7	Question 8
Molassiotis A	2019	☆	☆	☆	-	☆☆	☆	☆	☆
Chen C	2019	☆	☆	☆	-	☆	☆	☆	☆
Yamaguchi K	2018	☆	☆	☆	-	☆☆	☆	☆	☆
Hertz DL	2018	☆	☆	☆	-	☆☆	☆	☆	☆
Dolan ME	2017	☆	☆	☆	-	☆	☆	☆	☆
Pereira S	2016	☆	☆	☆	-	☆	☆	☆	☆
Ding XF	2015	☆	☆	☆	-	-	☆	☆	☆
Eckhoff L	2014	☆	☆	☆	-	☆	☆	☆	☆

Xue YJ	2013	☆	☆	☆	-	-	☆	☆	☆
