# Supplementary material for: Diabetes mellitus as a risk factor for chemotherapy-induced peripheral neuropathy: a meta-analysis
Source: Support Care Cancer. 2021 Jun 3;29(12):7461–9. doi: 10.1007/s00520-021-06321-7 (PMC8550712; doi:10.1007/s00520-021-06321-7)
Supplement: Supplementary file 4 — Table S4: Adjusted Newcastle–Ottawa scale (NOS) scores for the case-control studies (PDF 51 kb) [file 520_2021_6321_MOESM4_ESM.pdf]

Table S4: Adjusted Newcastle–Ottawa scale (NOS) scores for the case-control studies

Name	Year	Selection				Comparability	Outcome		
		Question 1	Question 2	Question 3	Question 4	Question 5	Question 6	Question 7	Question 8
Gaballah A	2018	☆	☆	☆	-	☆	☆	☆	☆
Song SJ	2017	☆	☆	☆	☆	☆	☆	☆	☆
Bao T	2016	☆	☆	☆	-	☆	☆	☆	☆
Tanishima H	2016	☆	☆	☆	-	☆	☆	☆	☆
Wang YQ	2016	☆	☆	☆	☆	☆☆	☆	☆	☆
Hershman, D. L	2016	☆	☆	☆	-	☆	☆	☆	☆
Shahriari-Ahmadi A	2015	☆	☆	☆	-	☆	☆	☆	☆
Kus T	2015	☆	☆	☆	☆	-	☆	☆	☆

de la Morena Barrio P	2015	☆	☆	☆	☆	☆☆	☆	☆	☆
Johnson C	2015	☆	☆	☆	-	-	☆	☆	☆
Wang XY	2013	☆	☆	☆	-	☆	☆	☆	☆
Hashimoto N	2012	☆	☆	☆	-	☆	☆	☆	☆
Kawakami K	2012	☆	☆	☆	-	☆	☆	☆	☆
Vincenzi B	2012	☆	☆	☆	-	☆	☆	☆	☆
Uwah AN	2012	☆	☆	☆	☆	☆	☆	☆	☆
Ramanathan, R. K	2010	☆	☆	☆	-	☆	☆	☆	☆
